# Sildenafil as an Emerging Analgesic: Mechanistic Insights and Therapeutic Potential in Pain Management

**DOI:** 10.1002/jbt.71155

**Published:** 2026-11-15

**Authors:** Radheshyam Pal, Utpal Bhui, Joy Das, Priya Chaudhary, Sumel Ashique, Bimlesh Kumar

**Affiliations:** ^1^ School of Pharmaceutical Sciences Lovely Professional University Phagwara India; ^2^ Centre for Herbal Pharmacology and Environmental Sustainability Chettinad Hospital and Research Institute, Chettinad Academy of Research and Education Kelambakkam India; ^3^ Department of Pharmaceutical Technology School of Health and Medical Sciences, Adamas University Kolkata India

**Keywords:** neuropathic pain, nitric oxide (NO)/cGMP signaling pathway, pain management, PDE‐5 inhibitor, sildenafil

## Abstract

Neuropathic pain is pain resulting from damage or disease affecting the peripheral or central nervous system. Chronic neuropathic pain is a major clinical concern and is frequently unresponsive to standard analgesics, so multimodal treatments have been suggested. There is substantial evidence supporting a role for the nitric oxide (NO)/cyclic guanosine monophosphate (cGMP) signaling pathway in nociceptive processing. It is unclear what the specific function of phosphodiesterase‐5 (PDE‐5) in the NO/cGMP pathway is in response to pain. In the present review, we aimed to evaluate potential peripheral interactions of sildenafil, a PDE‐5 inhibitor. Sildenafil is a specific and strong inhibitor of phosphodiesterase type 5 (PDE‐5). The enzyme PDE‐5 primarily degrades cGMP, an intracellular second messenger. Inhibition of cGMP breakdown results in higher intracellular cGMP concentrations, which are considered largely responsible for its therapeutic effect. This is a lacuna in the present literature, and this study seeks to provide an overall overview of the therapeutic potential of sildenafil in pain management. This realization of sildenafil's broader potential may lead to new therapeutic opportunities, the optimization of current strategies, and the expansion of its clinical use.

## Graphical abstract

Sildenafil as an emerging analgesic: The reuse of phosphodiesterase 5 (PDE5) inhibitors, particularly sildenafil, has become a promising approach to pain management.

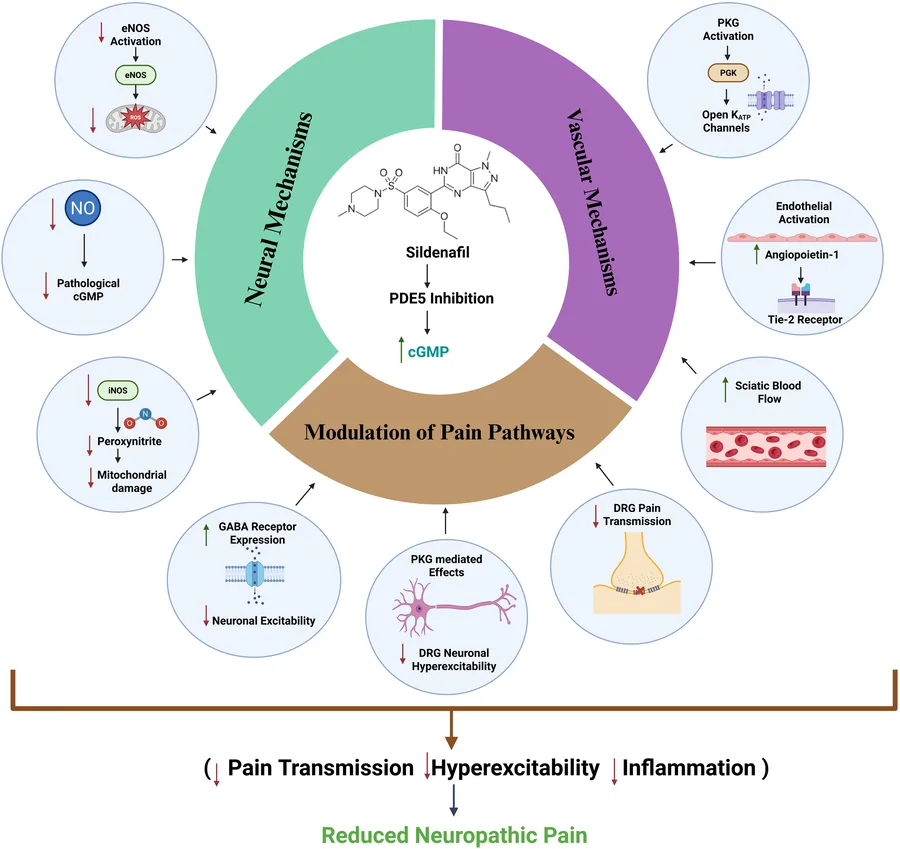

**Abbreviations**
AMPK: AMP‐activated protein kinase
AUC: total drug exposure
CCI: chronic constriction injury
cGMP: cyclic guanosine monophosphate
COX‐2: cyclooxygenase‐2
CREB/BDNF: cAMP response element‐binding protein/brain‐derived neurotrophic factor
CYP2C9: cytochrome P450 2C9
CYP3A4: cytochrome P450 3A4
eNOS: endothelial NOS
GABA: gamma‐aminobutyric acid
IASP: International Association for the Study of Pain
ICD‐11: International Classification of Diseases, 11th Revision
iNOS: inducible NOS
NADPH: nicotinamide adenine dinucleotide phosphate
NMDA: N‐methyl‐d‐aspartate
NO: nitric oxide
NO–cGMP: nitric oxide–cyclic guanosine monophosphate
NSAIDs: nonsteroidal anti‐inflammatory drugs
PDE5: phosphodiesterase 5
PI3K/Akt: phosphatidylinositol 3‐kinase/Akt is a protein kinase B
PKG: protein kinase G
sGC: soluble guanylate cyclase
SNL: spinal nerve ligation
SNRIs: serotonin and norepinephrine reuptake inhibitors
TCAs: tricyclic antidepressants
TRPV1: transient Receptor Potential Vanilloid 1
VAS: visual analog scale

## 1 Introduction

Chronic pain, identified as pain lasting more than 3 months, has now become a significant health issue around the globe with complex epidemiology and multivariate pathophysiology. The ICD‐11 criteria have 7 major types of chronic pain: primary, cancer‐related, orofacial, musculoskeletal, visceral, neuropathic, and post‐surgical/traumatic. The IASP additionally brought out the concept of nociplastic pain, characterized by disorders such as fibromyalgia, which is characterized by a change in central pain processing without indications of tissue or nerve damage [1, 2]. Chronic pain is a condition that affects up to 1 in 5 people worldwide, with significant individual and societal medical costs estimated up to US$635 billion annually in the U.S. alone. The prevalence depends on the area and the population. In America, around 35% of the population suffers from some kind of chronic pain, while in clinical investigations, 8%–10% of patients are often found to be in moderate‐to‐severe pain. In some nations, up to 55% of the population is affected. Higher frequency and worse outcomes are observed in vulnerable populations such as women and those with mental health comorbidities. Chronic pain arises from a variety of causes at the cellular level: nociceptive pain, caused by constant painful stimuli; neuropathic pain, caused by neural lesions or malfunction; and nociplastic pain, caused by abnormal central processing. Key processes include peripheral and central sensitization, wind‐up events in the spinal dorsal horn, incorrect activation of C‐fibers and maladaptive neuroplastic alterations. These changes lower pain thresholds and increase responses to painful and innocuous stimuli, resulting in symptoms such as allodynia and hyperalgesia [3].

Neuroimaging and proteomic studies show increased central sensory gain, neuroinflammation, and mitochondrial dysfunction in disorders such as fibromyalgia, indicating a convergence of nociplastic processes. First‐line medication can achieve just 30% pain reduction at best, and opioids, although often used, have potential risks of tolerance, dependence, endocrine disruption, and fatality [4]. These difficulties highlight the necessity of multimodal approaches that integrate pharmaceutical, psychological, and interventional strategies. To find more secure and efficient medications, there is increasing emphasis on biochemical systems such as AMPK, NO‐cGMP, and inflammatory signaling [5]. The pharmaceutical treatments for persistent pain that are available at present include anticonvulsants, antidepressants, opioids, and NSAIDs, all of which have safety concerns and inadequate efficacy. NSAIDs are helpful for inflammatory pain, but excessive amounts of them might cause hepatorenal damage, gastrointestinal ulcers, renal failure, and increased risk of heart disease (especially in elderly patients or those on polypharmacy) [6, 7]. Despite their potency, opioids only provide temporary relief, are not superior to NSAIDs, and may even be less efficient in treating pain that is not related to cancer. There is no proof that they enhance function over time. Tolerance, physical dependence, hypogonadism, depressive disorders, and paradoxical opioid‐mediated hyperalgesia are all part of their adverse nature, which can make persistent pain conditions worse. There is a problem of opioid usage and variation in prescribing procedures as a result of the growing use and the associated risks [8]. Antidepressants (such as SNRIs and TCAs) and anticonvulsants (such as gabapentin and pregabalin) that focus on neuropathic pathways are examples of adjuvant medications; however, their benefits are minimal, and there are pharmacokinetic issues. Antidepressants frequently cause depression, gastrointestinal distress, or cardiovascular complications and only slightly reduce pain (e.g., ~0.6/10 on the VAS for osteoarthritis). Anticonvulsants can interact with P450 enzymes, complicating polypharmacy management. They also cause cognitive impairment, nausea, and withdrawal issues [9]. Although topical analgesics are more effective, they are insufficient to treat widespread chronic pain since they only provide local and occasionally temporary relief. This necessitates the development of novel analgesics with superior safety benefits within a therapeutic window and that are not mechanistically limited, which is why sildenafil is being investigated for pain relief [10]. Preclinical investigation has shown that reusing phosphodiesterase 5 (PDE5) inhibitors, particularly sildenafil, is a feasible approach to pain management. PDE5 inhibitors are being studied for their analgesic and neuroinflammatory effects, following their initial identification for their vasodilatory and endothelium‐protective properties in pulmonary hypertension and erectile dysfunction [11].

## 2 Pharmacological Profile of Sildenafil

### 2.1 Chemical Structure and Mechanism of Action

Sildenafil was identified chemically as [4‐ethoxy‐3‐ (6,7‐dihydro‐1‐methyl‐7‐oxo‐3‐propyl‐1H‐pyrazolo [4,3‐d] pyrimidin‐5‐yl)phenylsulphonyl] 4‐methylpiperazine. Its methylpiperazine moiety, ethoxy‐phenyl group, and pyrazolopyrimidinone core are responsible for its potency and selectivity as a phosphodiesterase 5 (PDE5) inhibitor. X‐ray crystallography and subsequent molecular docking reveal the stiff coplanar structure of sildenafil within PDE5. This conformation engages residues including Leu804, Val782, Tyr612, and Phe820, which underpin its high‐binding affinity (IC_50_ ≈ 3–5 nM) [12]. Sildenafil's minor binding to PDE6, a retinal enzyme, underlies its adverse visual effects (at micromolar levels, disrupting phototransduction), despite its chemical resemblance to other members of the PDE family. PDE1‐4 have selectivity ranging from 80 to 19,000 times.

### 2.2 Pharmacokinetics and Pharmacodynamics

The widely recognized pharmacokinetic and pharmacodynamic characteristics of sildenafil, which support its authorized uses and efficacy in pain management, have been shown in recent studies and academic reviews. Oral sildenafil reaches maximal plasma concentration (Tmax) in 30 to 120 min, typically 60 min in those who are fasting [13]. The typical absorption is approximately 40%. A high‐fat breakfast reduced peak concentration (Cmax) by approximately 29%–30% and prolonged the absorption phase by about 60 min, but had no effect on total drug exposure (AUC). The drug's wide tissue penetration is indicated by its enormous volume of distribution (~105 L). At all treatment concentrations, the percentage bound to standard plasma proteins remains at 96% [14]. About 50% of sildenafil's PDE5‐inhibitory potency and 20%–40% of its medical benefits are attributed to the potent N‐desmethyl metabolite (UK 103,320). The liver‐specific CYP3A4 is primarily responsible for this drug's metabolism, with CYP2C9 playing a smaller role. According to the dose schedule, sildenafil and its analog have an ultimate half‐life of roughly 3 to 5 h. About 80% of the dosage is eliminated by the liver, while about 13% is eliminated in urine [15]. Patient characteristics primarily affect sildenafil metabolism. In elderly individuals (≥ 65 years), clearance is slowed, and plasma levels of both the parent and the metabolite increase by ~84%–107%, with free drug exposure increasing by ~45%–57%.

### 2.3 Safety and Adverse Effect Profile

Sildenafil has been shown to be generally well tolerated in clinical studies and post‐marketing surveillance, with most side effects being mild to moderate and temporary. Headache (12%–20%), flushing (10%–15%), dyspepsia, nasal congestion, back discomfort, myalgia and visual problems such as temporary color changes or impaired vision are common, reflecting vasodilatory effects and cross‐reactivity with PDE6 in the retina [14, 16]. Large pooled analyses (> 14,000 men in placebo‐controlled trials and post‐marketing data) confirm no causal association between sildenafil (50–100 mg) and major cardiovascular events, hearing loss, non‐arteritic ischemic optic neuropathy (NAION), or priapism at allowed levels [14]. Serious adverse events are rare but can occur, especially in high‐risk populations. Other reports have suggested a relationship between NAION and older males. Sudden hearing loss has also been reported in post‐marketing use, and FDA label warnings have been issued, but no proven causality exists [17]. Rare problems are hypotension and priapism, most commonly seen at elevated dosages or when sildenafil is co‐administered with alpha blockers, nitrates, or powerful ritonavir. These interactions can cause a significant increase in sildenafil levels and are prohibited [18]. Sildenafil at therapeutic doses presents a favorable safety profile, with low discontinuation rates (~2%–3%) in long‐term studies and no emergence of new safety signals even in elderly or systemically ill patients [14].

## 3 Mechanisms of Pathological Pain Cascade and its Modulation By Sildenafil

Peripheral Nerve Injury causes an over‐activation of pre‐ and post‐synaptic N‐methyl‐d‐aspartate receptors (NMDAR), Voltage‐Gated Calcium Channels (VGCC), and Transient Receptor Potential (TRP) channels. This results in a huge increase in intracellular Ca^2+^ concentration. The increase in intracellular calcium activates inducible nitric oxide synthase (iNOS) and endothelial nitric oxide synthase (eNOS). iNOS activation leads to the generation of superoxide, which promotes the accumulation of Reactive Oxygen Species (ROS) and causes inflammation. Simultaneously, iNOS activation generates Peroxynitrite (ONOO‐), leading to structural damage to mitochondria. At the same time, Nitric Oxide (NO) directs soluble Guanylyl Cyclase (sGC) with an associated pathological rise in cGMP levels. This stimulates Protein Kinase G (PKG) and Protein Kinase C gamma (PKCγ), leading to increased downstream neuronal excitability and consequent neuropathic pain (Figure 1) [5].

**Figure 1:**
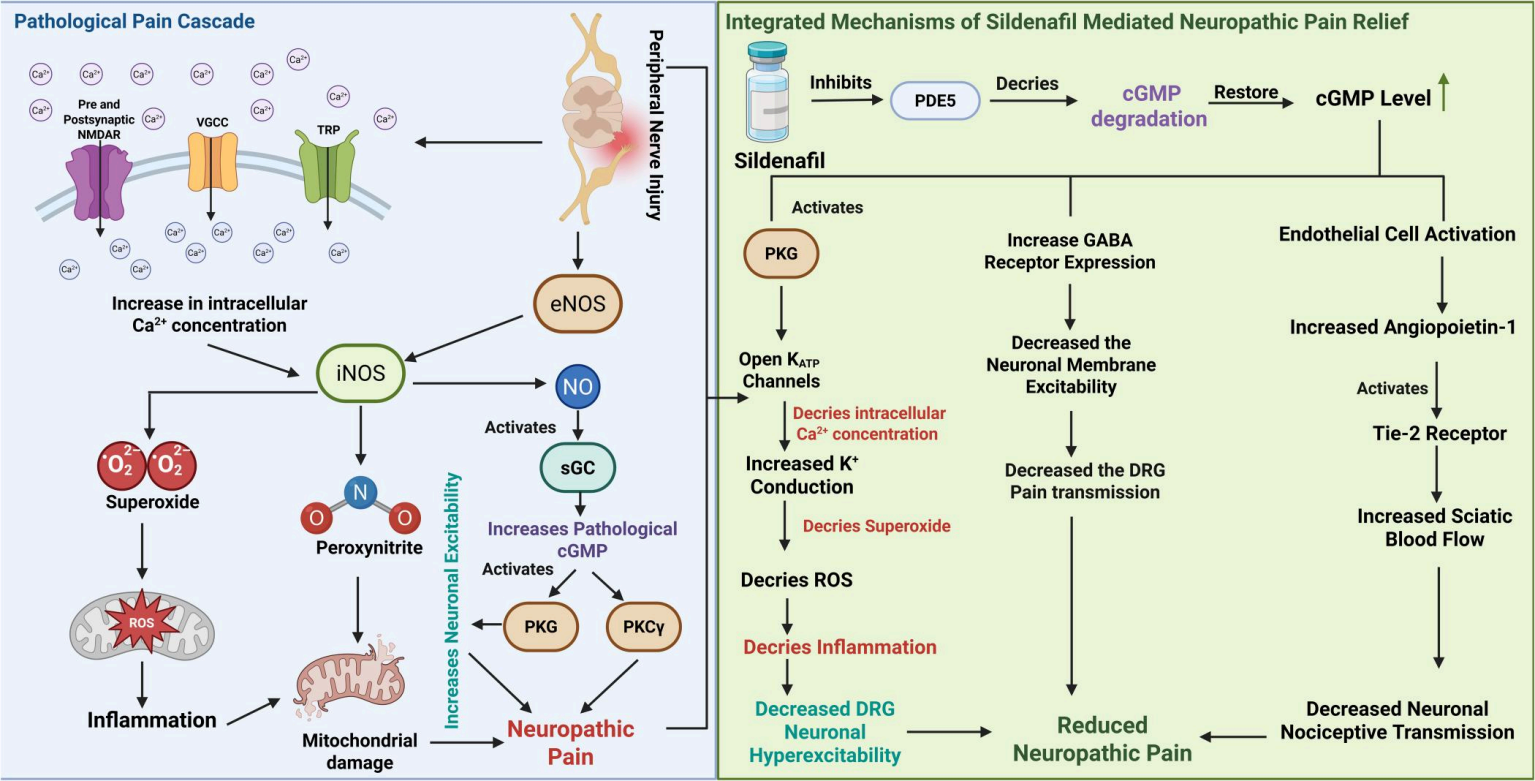
Pathophysiological cascade of neuropathic pain and mechanisms of action of sildenafil in therapy. Massive intracellular Ca^2+^ influx induced by peripheral nerve injury leads to oxidative stress (iNOS‐mediated superoxide and peroxynitrite production), mitochondrial damage, and abnormal cGMP/PKG/PKCγ signaling, culminating in neuropathic pain. Sildenafil inhibits PDE5, preventing cGMP breakdown, thereby coordinating three pain‐relieving pathways: PKG/K_ATP_ axis, PKG activation, and K_ATP_ channel opening to decrease Ca^2+^ influx, ROS/superoxide levels, and DRG neural hyperexcitability. GABAergic Modulation: Augmentation of GABA receptor expression to diminish cellular membrane excitability and transmission of pain from the dorsal root ganglion (DRG). Neurovascular Restoration: Engages endothelial Angiopoietin‐1/Tie‐2 signaling to improve sciatic blood flow and reduce nociceptive transmission. cGMP, cyclic guanosine monophosphate; DRG, dorsal root ganglion; eNOS/iNOS, endothelial/inducible nitric oxide synthase; GABA, gamma‐aminobutyric acid; KATP, sensitive potassium channel; NMDAR, N‐methyl‐d‐aspartate receptor; NO, nitric oxide; PDE5, phosphodiesterase‐5; PKG/PKCγ, protein kinase G/protein kinase C gamma; ROS, reactive oxygen species; sGC, soluble guanylyl cyclase; TRP, transient receptor potential; VGCC, voltage‐gated calcium channel.

Sildenafil is a strong and specific phosphodiesterase type 5 (PDE5) inhibitor [19]. Phosphodiesterases are classified into several classes; however, only three of them (PDE5, PDE6, and PDE9) selectively hydrolyze cyclic guanosine monophosphate (cGMP) rather than cyclic adenosine monophosphate (cAMP) [20]. Because sildenafil inhibits PDE5, the breakdown of cGMP is slowed, and its homeostatic levels are restored or even raised [21, 22]. Through three functional pathways, this elevation prevents the cascade of pathological pain. **The PKG/KATP Channel Axis:** ATP‐sensitive potassium (KATP) channels are encouraged to open when PKG is activated by elevated cGMP. Increased K+ conduction is enabled by this modulation of homeostasis, which directly lowers the intracellular Ca2+ concentration. When calcium levels return to normal, this pathway lowers ROS, superoxide, and inflammation. By lowering DRG neuronal hyperexcitability, the resolution of oxidative and inflammatory pathways directly lessens neuropathic pain [23]. **GABAergic Modulation:** Higher cGMP causes an increase in GABA receptor expression. Decreased neuronal membrane excitability. Enhanced expression of GABA receptors. This hyperpolarization inhibits aberrant signaling, resulting in reduced DRG pain transmission and reduced neuropathic pain [24]. **Neurovascular/Angiopoietin‐1 Flow Restoration:** Sildenafil‐induced cGMP accumulation activates endothelial cells [25]. This results in the production and upregulation of Angiopoietin‐1, which then binds and activates the Tie‐2 receptors. Tie‐2 receptor activation promotes microvascular alterations that result in increased sciatic blood flow [26]. Decreased neuronal nociceptive transmission and neuropathic pain. Terminal alleviation through improved tissue perfusion and neurovascular function (Figure 1).

NO (nitric oxide) is also involved in the pathophysiology of Diabetes Neuropathy (DN) through activation of iNOS induced by chronic inflammation. NO suppresses smooth muscle cell proliferation and platelet aggregation [27]. Several studies indicate reciprocal interaction between NO and prostaglandin pathways and reveal that high levels of NO accelerate the course of DN [28, 29]. Thus, NO acts directly on COX expression and indirectly on PG (prostaglandin) synthesis. Conversely, COX isoforms do modulate NO production via arachidonic acid or its metabolites. NO regulates COX‐2 activity through post‐transcriptional and translational mechanisms, resulting in increased macrophage PG synthesis [30]. Inactivation of iNOS by inhibiting the NF‐κB signaling pathway after NO production by constitutive NOS. One of the signaling routes that induces iNOS production in response to external mediators such as endotoxin and TNF‐α is NF‐κB signaling. By inhibiting NF‐κB, NO suppresses COX‐2, as NF‐κB is a potent inducer of COX‐2. Various peripheral cell types release NO in response to inflammation. Once expressed, iNOS will generate significant levels of NO for a long period [31]. Metformin demonstrated potential to reduce iNOS expression and NO production by downregulating NF‐κB translocation. Sildenafil downregulated iNOS expression by inhibiting its protein and mRNA levels. In one of the studies, after 14 days of treatment, nitrite levels in brain tissues were markedly reduced in the S3 + M500 and S2.5 + M250 combinations compared with the diabetic control group. All diabetic groups treated with gabapentin and the three sildenafil–metformin combinations showed a remarkable reduction in nitrite levels in liver tissue compared with the diabetic control group, suggesting lower NOS activity. To summarize, this study, for the first time, demonstrated that the sildenafil–metformin combination effectively alleviated the symptoms of diabetic neuropathy [32].

## 4 Pre‐Clinical Evidence of Sildenafil in the Pain Model

Research investigations have highlighted the analgesic properties of sildenafil, which can modulate the nitric oxide (NO)/cyclic guanosine monophosphate (cGMP) pathway, and there is evidence that these compounds, likely PDE5 inhibitors or NO donors, have broad efficacy across pain models. Preclinical studies utilizing models of neuropathic pain have shown analgesic effects, as shown in Table 1.

**Table 1:** Summary of preclinical studies on sildenafil in different pain models.

S. No.	Pain model (Species)	Dose and route	Key findings	Proposed mechanism(s)	References
1.	Neuropathic pain—chronic constriction injury (rat)	100 and 200 μg/rat; intrathecal	Marked reduction in pain threshold	It showed substantial activation of the NO‐cGMP pathway in the chronic constriction injury model of neuropathic pain. Increased cGMP levels may lead to PKG‐I activation and its up‐regulation, which can aggravate the hyperalgesic response.	[33]
2.	Inflammation and oxidative stress in pelvic ganglia neurones after bilateral cavernosal nerve injury (rat)	20 mg/kg; oral	Reduce the synthesis of harmful factors and may upregulate the expression of positive factors in the MPG, thereby relieving neuropathic pain, enhancing neuroprotection, and favoring neuron regeneration.	Showed that BCNR in Fisher‐344 rats up‐regulated cytokines and oxidative stress factors that were normalized after the treatment with sildenafil.	[34]
3.	Intrathecal combination with morphine (rat) ‐ formalin test	1, 3, 10, and 30 µg; intrathecal	Improved morphine analgesia	In phase 1 and phase 2 of the formalin test, sildenafil and morphine produced an antinociceptive effect. Isobolographic research demonstrated an additive interaction after intrathecal administration of sildenafil and morphine in both stages.	[35]
4.	Sildenafil and clonidine in the formalin test on rats	1, 3, 10, and 30 µg; intrathecal	Improved clonidine‐induced analgesia	The impact of clonidine is guanylate cyclase‐independent, whereas the antinociceptive effect of sildenafil can be modified by spinal alpha‐2 adrenoceptors.	[36]
5.	Peripheral neuropathy in diabetic type ii mice	10 mg/kg, oral	Sildenafil ameliorates long‐term peripheral neuropathy in middle‐aged diabetic mice, and its alleviation of neurovascular dysfunction likely contributes to improved nerve function.	The Ang1/Tie2 signaling pathway plays a crucial role in the restorative processes.	[26]
6.	Alloxan‐induced diabetic neuropathy in rats	1.5, 2.5, and 3 mg/kg, oral	Reduced inflammation, prevented an increase in NOS activity, and protected against glutathione depletion.	Pro‐inflammatory cytokines (IL‐6 and TNF‐α) and changes in nitrites and thiols concentrations.	[37]
7.	Haemodynamics and neuropathic pain in rats	1, 3, 10, and 30 mg/kg bw; intraperitoneal	Dose‐dependent antinociception	Inhibition of PDE‐5 leads to an accumulation of cGMP, which then causes the release of GABA and activation of GABAa/GABAb receptors. This ultimately blocks pain.	[24]
8.	Peripheral antinociptive effect of ellagic acid alone and in conjunction with sildenafil in the rat formalin test	25 or 50 mcg/paw, intraperitoneal	Potentiates the peripheral antinociceptive effect of ellagic acid	In both phases of the test, local administration of ellagic acid and sildenafil was dose‐dependently associated with increased nociception.	[38]
9.	Co‐administration against neuropathic pain generated by chronic constriction injury in rats	5‐10 mg/kg; intraperitoneal	Pronounced Additive Analgesia	Fluoxetine increases the activity of descending monoaminergic inhibitory pain pathways by selectively inhibiting serotonin reuptake. Sildenafil inhibits PDE‐5 and prevents the breakdown of intracellular cyclic guanosine monophosphate (cGMP).	[39]
10.	Neuropathy in rats induced by alcohol	5 and 10 mg/kg; intraperitoneal	Reduce symptoms of alcohol‐induced neuropathic pain	Both drugs, when given individually at their low as well as high dosages, could ameliorate the symptoms of alcohol caused neuropathic pain. Co‐administration of curcumin and sildenafil at their lower levels was observed to considerably improve nerve function, biochemical parameters, and histopathological parameters compared with their separate administration.	[40]
11.	Streptozotocin‐induced diabetic mouse model	20 mg/kg, intragastric	Therapeutic efficacy of islet transplantation	The results demonstrated that islet transplantation could repair sciatic nerve injury in diabetic mice, and that sildenafil could enhance its therapeutic effect. Furthermore, the combination of sildenafil and islet cells dramatically increased MBP, BDNF, and mTOR/S6K1 levels in RSC96 cells under high‐glucose conditions.	[41]
12.	Mouse model of peripheral nerve injury	5 ml/kg, intraperitoneal	PDE4i and PDE5i reduce mechanical allodynia following peripheral nerve damage.	Pharmacological and knock‐out techniques were used to show the preferential implication of delta opioid receptors in the action of the PDE4i rolipram and of both mu and delta opioid receptors in the action of the PDE5i sildenafil.	[42]

## 5 Clinical Trials of Sildenafil in Pain

Two clinical trials explored the therapeutic potential of sildenafil in pain‐related conditions. An independent open‐label, non‐randomized dose‐finding trial (NCT00194909) investigated sildenafil (Viagra) in patients with painful neuropathy secondary to diabetes mellitus. The primary outcome was the change in the weekly average of the 11‐point Likert pain scale over 8 weeks. The purpose of this trial was to determine whether sildenafil was effective and tolerable in chronic neuropathic pain, and provided evidence supporting a change in pain intensity scores from baseline to week 8, although this does not have the study rigor seen in randomized studies [43]. These studies suggest a new use of sildenafil in chronic pain management in addition to erectile dysfunction, and larger placebo‐controlled trials are needed in this area. The second trial involves a 40‐year‐old male with very severe neuropathic pain following a lumbar nephrectomy for renal neoplasia. First‐, second‐, or third‐line medications administered at standard doses did not provide sufficient pain relief. Sildenafil (6 months) has been effective in controlling the pain, notably reducing its severity and significantly improving the patient's functional restriction. Sildenafil has achieved a considerable decrease in pain intensity and is emerging as a therapeutic option for neuropathic pain uncontrolled by standard therapy [44].

## 6 Challenges, Limitations, and Safety Considerations

By altering the nitric oxide–cGMP signaling pathway, sildenafil is a promising analgesic under development. However, sildenafil's widespread medical use in the treatment of pain is hampered by a number of issues and disadvantages. To ensure the safe transfer of experimental studies into the clinical setting, these features must be carefully considered. The absence of solid clinical data is one of the main challenges. Preclinical studies in animal models of migraine, inflammatory, and neuropathic pain have provided the majority of the information on sildenafil's analgesic properties thus far [19]. These models may not accurately capture the intricate pathological mechanisms underlying human pain conditions, even though they offer mechanistic insight. To determine the efficacy, optimal dosage, and long‐term effects of sildenafil across different pain categories, extensive randomized clinical trials must be conducted. The use of analgesics is not FDA‐approved, which causes issues like ambiguous dosage guidelines and unidentified therapeutic windows. Although higher concentrations may be more effective at reducing pain, there may be a higher chance of serious systemic and cardiovascular effects. Additionally, the safety and effectiveness of pharmaceuticals may be significantly affected by interindividual differences in their metabolism, driven by CYP3A4 genetic variants or drug‐drug interactions [45]. When thinking about using sildenafil to treat chronic pain, safety issues are the most crucial. Common issues that can impede long‐term medication adherence include visual abnormalities, flushing, headaches, dyspepsia, and nasal congestion. Cardiovascular problems, priapism, and hypotension are more serious issues, especially in people with pre‐existing heart conditions or those taking nitrate‐based medications. In clinical pain procedures, sildenafil administration entails careful patient evaluation, dose modifications, and vigilant monitoring [19]. The possibility of drug interactions is another major limitation. Anticonvulsants, antidepressants, non‐steroidal anti‐inflammatory drugs (NSAIDs), opioids, and various treatment regimens may be prescribed to people with chronic pain [46]. These medications may change pharmacokinetics or increase toxicity, and their interactions with sildenafil are poorly understood. However, since sildenafil is primarily prescribed for erectile dysfunction, the idea of using it as an analgesic could worry patients and physicians. Adoption of standard pain management recommendations may be impacted by inadequate evidence for off‐label medical treatments, which may result in medico‐legal problems.

## 7 Future Research

The potential use of sildenafil as an analgesic medication opens up new possibilities in pain management because the effects of traditional medications are temporary. However, more research is necessary to evaluate its therapeutic relevance, improve treatment strategies, and ensure patient quality of life, as our current understanding remains incomplete. There are many opportunities and avenues for research to advance this field. In an attempt to validate the preclinical findings demonstrating sildenafil's analgesic benefits, large‐scale randomized controlled trials (RCTs) are a top priority. RCTs should be conducted in a diverse population with discomfort related to carcinoma, inflammation, and cognition to evaluate the efficacy of sildenafil across these possible causes. Additionally, the trials will help determine the appropriate dosing schedules, treatment duration, and therapeutic goals. Further mechanistic investigation is also required. Investigating sildenafil's interactions with other molecular targets (such as glial cell activity, neurotransmitter release, or ion channels) may reveal novel mechanisms underlying its analgesic effects. The development of next‐generation PDE5 inhibitors with enhanced efficacy and fewer side effects, especially intended for the medical treatment of pain, may benefit from these findings. The investigation of combination therapies is another possible field of study. Sildenafil may work in concert with opioids, NSAIDs, or adjuvant medications like gabapentin and antidepressants to treat chronic pain, which is rarely accomplished with a single medication.

## 8 Conclusion

Sildenafil is an effective medication with an acceptable safety and tolerability profile. It is also more effective across numerous subpopulations but less effective in the designated difficult‐to‐treat groups. Studies have shown that sildenafil may act on the nociceptive network in pain management. It can lengthen activation latencies for pain in animal models of neurological pain (e.g., pain induced by diabetes), potentially mitigating chronic pain. The primary mechanism is to activate the cGMP system, which is involved in the processing of pain signals. Sildenafil's impact on this pathway might reduce pain responses, thereby providing a rationale for examining its potential for treating chronic pain in clinical settings. Traditionally advised for erectile dysfunction, sildenafil shows preliminary data that it may serve as a possible alternative or supplementary option for severe, treatment‐resistant neuropathic pain. However, due to the significant risk of bias or placebo effect in open‐label trials and single‐case reports, validation in large‐scale, randomized, placebo‐controlled clinical trials is necessary for broad clinical acceptance. Furthermore, new results from basic and clinical studies suggest a potential role for several other chronic diseases in the future.

## Author Contributions

**Radheshyam Pal:** writing – original draft, resources. **Utpal Bhui:** resources, writing – original draft. **Joy Das:** conceptualization, software, formal analysis. **Priya Chaudhary:** conceptualization, software, formal analysis. **Sumel Ashique:** supervision, writing – original draft, validation. **Bimlesh Kumar:** validation, supervision, writing – review and editing.

## Funding

The authors have nothing to report.

## Ethics Statement

The authors have nothing to report.

## Conflicts of Interest

The authors declare no conflicts of interest.

## Data Availability

The data presented in this study are available on reasonable request from the corresponding author.

## References

[1] A. Barke , B. Korwisi , and W. Rief , “Chronic Pain in the ICD‐11: New Diagnoses That Clinical Psychologists Should Know About,” Clinical Psychology in Europe 4, no. Spec Issue (2022): e9933. 10.32872/cpe.9933 PMC9881113 36760323

[2] Y. L. Zhang , X. C. Wu , X. Y. Chen , F. Gao , and J. Wang , “Global and Regional Burden, Temporal Trends, and Projections of Chronic Pain From 1990 to 2032, and Its Association With Cardiovascular Diseases: Analyses Based on Global Burden of Diseases Study 2021,” Frontiers in Public Health 13 (2025): 1636949. 10.3389/fpubh.2025.1636949 PMC12660301 41323632

[3] F. F. Pandelani , S. L. N. Nyalunga , M. M. Mogotsi , and V. B. Mkhatshwa , “Chronic Pain: Its Impact on the Quality of Life and Gender,” Frontiers in Pain Research 4 (2023): 1253460. 10.3389/fpain.2023.1253460 PMC10534032 37781217

[4] A. T. Leerling , M. Niesters , M. Flendrie , et al., “Neuropathic and Nociplastic Pain Profiles Are Common in Adult Chronic Nonbacterial Osteitis (CNO),” Calcified Tissue International 114, no. 6 (2024): 603–613. 10.1007/s00223-024-01214-3 PMC11090977 38627292

[5] D. Y. Li , S. J. Gao , J. Sun , et al., “Targeting the Nitric Oxide/cGMP Signaling Pathway to Treat Chronic Pain,” Neural Regeneration Research 18, no. 5 (2023): 996–1003. 10.4103/1673-5374.355748 PMC9827765 36254980

[6] N. M. Alorfi , “Pharmacological Methods of Pain Management: Narrative Review of Medication Used,” International Journal of General Medicine 16 (2023): 3247–3256. 10.2147/IJGM.S419239 PMC10402723 37546242

[7] Y. Shi and W. Wu , “Multimodal Non‐Invasive Non‐Pharmacological Therapies for Chronic Pain: Mechanisms and Progress,” BMC Medicine 21, no. 1 (2023): 372. 10.1186/s12916-023-03076-2 PMC10542257 37775758

[8] A. A. Altawili , M. A. Altawili , A. H. Alzarar , et al., “Adverse Events of the Long‐Term Use of Opioids for Chronic Non‐Cancer Pain: A Narrative Review,” Cureus 16, no. 1 (2024): e51475. 10.7759/cureus.51475 PMC10830133 38298287

[9] J. Bernatoniene , A. Sciupokas , D. M. Kopustinskiene , and K. Petrikonis , “Novel Drug Targets and Emerging Pharmacotherapies in Neuropathic Pain,” Pharmaceutics 15, no. 7 (2023): 1799. 10.3390/pharmaceutics15071799 PMC10384314 37513986

[10] M. Carrillo , J. Yingst , W. J. Tuan , D. Giampetro , J. E. Nyland , and A. E. Zgierska , “Problems Accessing Pain Care, and the Adverse Outcomes Among Adults With Chronic Pain: A Cross‐Sectional Survey Study,” Pain Management 15, no. 2 (2025): 81–91. 10.1080/17581869.2025.2463865 PMC11853623 39936847

[11] A. K. ElHady , D. S. El‐Gamil , M. Abdel‐Halim , and A. H. Abadi , “Advancements in Phosphodiesterase 5 Inhibitors: Unveiling Present and Future Perspectives,” Pharmaceuticals 16, no. 9 (2023): 1266. 10.3390/ph16091266 PMC10536424 37765073

[12] M. T. M. Nemr , M. A. Abdelaziz , M. Teleb , A. E. Elmasry , and Y. A. A. M. Elshaier , “An Overview on Pharmaceutical Applications of Phosphodiesterase Enzyme 5 (PDE5) Inhibitors,” Molecular Diversity 29, no. 5 (2025): 4765–4785. 10.1007/s11030-024-11016-2 PMC12454544 39592536

[13] D. J. Nichols , G. J. Muirhead , and J. A. Harness , “Pharmacokinetics of Sildenafil After Single Oral Doses in Healthy Male Subjects: Absolute Bioavailability, Food Effects and Dose Proportionality,” supplement, British Journal of Clinical Pharmacology 53 Suppl 1, no. 5 (2002): 5S–12S. 10.1046/j.0306-5251.2001.00027.x PMC1874258 11879254

[14] F. Giuliano , G. Jackson , F. Montorsi , A. Martin‐Morales , and P. Raillard , “Safety of Sildenafil Citrate: Review of 67 Double‐Blind Placebo‐Controlled Trials and the Postmarketing Safety Database,” International Journal of Clinical Practice 64, no. 2 (2010): 240–255. 10.1111/j.1742-1241.2009.02254.x PMC2810448 19900167

[15] A. Ouranidis , A. Tsiaxerli , E. Vardaka , et al., “Sildenafil 4.0—Integrated Synthetic Chemistry, Formulation and Analytical Strategies Effecting Immense Therapeutic and Societal Impact in the Fourth Industrial Era,” Pharmaceuticals 14, no. 4 (2021): 365. 10.3390/ph14040365 PMC8071249 33920975

[16] E. Ausó , V. Gómez‐Vicente , and G. Esquiva ,“Visual Side Effects Linked to Sildenafil Consumption: An Update,” Biomedicines 9, no. 3 (2021): 291, 10.3390/biomedicines9030291. PMC7998971 33809319

[17] M. L. Salvetat , F. Pellegrini , L. Spadea , C. Salati , and M. Zeppieri , “Non‐Arteritic Anterior Ischemic Optic Neuropathy (NA‐AION): A Comprehensive Overview,” Vision 7, no. 4 (2023): 72. 10.3390/vision7040072 PMC10661278 37987292

[18] H. Chamsi‐Pasha , “Sildenafil (Viagra) and the Heart,” Journal of Family and Community Medicine 8, no. 2 (2001): 63–66. PMC3437061 23008645

[19] C. Pușcașu , A. Zanfirescu , S. Negreș , and O. C. Șeremet , “Exploring the Multifaceted Potential of Sildenafil in Medicine,” Medicina 59, no. 12 (2023): 2190. 10.3390/medicina59122190 PMC10744870 38138293

[20] Q. Saikia , A. Hazarika , and R. Mishra , “A Review on the Pharmacological Importance of PDE5 and Its Inhibition to Manage Biomedical Conditions,” Journal of Pharmacology and Pharmacotherapeutics 13, no. 3 (2022): 246–257.

[21] S. Nandi , P. Kumar , S. A. Amin , T. Jha , and S. Gayen , “First Molecular Modelling Report on Tri‐Substituted Pyrazolines as Phosphodiesterase 5 (PDE5) Inhibitors Through Classical and Machine Learning Based Multi‐QSAR Analysis,” SAR and QSAR in Environmental Research 32, no. 11 (2021): 917–939. 10.1080/1062936X.2021.1989721 34727793

[22] N. K. Jain , C. S. Patil , A. Singh , and S. K. Kulkarni , “Sildenafil‐Induced Peripheral Analgesia and Activation of the Nitric Oxide‐Cyclic GMP Pathway,” Brain Research 909, no. 1–2 (2001): 170–178. 10.1016/s0006-8993(01)02673-7 11478933

[23] T. Kawano , V. Zoga , M. Kimura , et al., “Nitric Oxide Activates ATP‐Sensitive Potassium Channels in Mammalian Sensory Neurons: Action by Direct S‐Nitrosylation,” Molecular Pain 5 (2009): 1744‐8069‐5‐12. 10.1186/1744-8069-5-12 PMC2673211 19284878

[24] L. J. Huang , M. H. Yoon , J. I. Choi , W. M. Kim , H. G. Lee , and Y. O. Kim , “Effect of Sildenafil on Neuropathic Pain and Hemodynamics in Rats,” Yonsei Medical Journal 51, no. 1 (2010): 82–87. 10.3349/ymj.2010.51.1.82 PMC2799976 20046518

[25] C. Pușcașu , S. Negreș , C. E. Zbârcea , et al., “Evaluating the Antihyperalgesic Potential of Sildenafil‐Metformin Combination and Its Impact on Biochemical Markers in Alloxan‐Induced Diabetic Neuropathy in Rats,” Pharmaceuticals 17, no. 6 (2024): 783. 10.3390/ph17060783 PMC11206800 38931450

[26] L. Wang , M. Chopp , A. Szalad , et al., “Sildenafil Ameliorates Long Term Peripheral Neuropathy in Type II Diabetic Mice,” PLoS One 10, no. 2 (2015): e0118134. 10.1371/journal.pone.0118134 PMC4331563 25689401

[27] J. Satoh , S. Yagihashi , and T. Toyota , “The Possible Role of Tumor Necrosis Factor‐α in Diabetic Polyneuropathy,” Journal of Diabetes Research 4, no. 2 (2003): 65–71. 10.1155/EDR.2003.65 PMC2478597 14630568

[28] A. Ozkul , M. Ayhan , C. Yenisey , A. Akyol , E. Guney , and F. A. Ergin , “The Role of Oxidative Stress and Endothelial Injury in Diabetic Neuropathy and Neuropathic Pain,” Neuroendocrinology Letters 31, no. 2 (2010): 261–264. 20424576

[29] V. Ramakrishna and R. Jailkhani , “Oxidative Stress in Non‐Insulin‐Dependent Diabetes Mellitus (NIDDM) Patients,” Acta Diabetologica 45, no. 1 (2008): 41–46. 10.1007/s00592-007-0018-3 17924055

[30] H. Togashi , M. Sasaki , E. Frohman , et al., “Neuronal (Type I) Nitric Oxide Synthase Regulates Nuclear Factor ϰ B Activity and Immunologic (Type II) Nitric Oxide Synthase Expression,” Japanese Journal of Pharmacology 75 (1997): 6. 10.1073/pnas.94.6.2676 PMC20148 9122255

[31] T. Purwata , “High TNF‐Alpha Plasma Levels and Macrophages iNOS and TNF‐Alpha Expression as Risk Factors for Painful Diabetic Neuropathy,” Journal of Pain Research 4 (2011): 169–175. 10.2147/JPR.S21751 PMC3141833 21811392

[32] C. Pușcașu , A. Ungurianu , O. C. Șeremet , C. Andrei , P. Mihai , and S. Negreș , “The Influence of Sildenafil–Metformin Combination on Hyperalgesia and Biochemical Markers in Diabetic Neuropathy in Mice,” Medicina 59, no. 8 (2023): 1375. 10.3390/medicina59081375 PMC10456948 37629665

[33] C. S. Patil , S. V. Padi , V. P. Singh , and S. K. Kulkarni , “Sildenafil Induces Hyperalgesia via Activation of the NO‐cGMP Pathway in the Rat Neuropathic Pain Model,” Inflammopharmacology 14, no. 1 (2006): 22–27. 10.1007/s10787-006-1511-y 16835709

[34] L. Garcia , S. Hlaing , R. Gutierrez , et al., “Sildenafil Attenuates Inflammation and Oxidative Stress in Pelvic Ganglia Neurons After Bilateral Cavernosal Nerve Damage,” International Journal of Molecular Sciences 15, no. 10 (2014): 17204–17220. 10.3390/ijms151017204 PMC4227157 25264738

[35] M. H. Yoon , K. D. Park , H. G. Lee , et al., “Additive Antinociception Between Intrathecal Sildenafil and Morphine in the Rat Formalin Test,” Journal of Korean Medical Science 23, no. 6 (2008): 1033. 10.3346/jkms.2008.23.6.1033 PMC2610640 19119449

[36] M. H. Yoon , C. M. Kim , H. G. Lee , et al., “Synergistic Antinociception of Intrathecal Sildenafil With Clonidine in the Rat Formalin Test,” Pharmacology, Biochemistry and Behavior 92, no. 4 (2009): 583–588. 10.1016/j.pbb.2009.02.007 19254741

[37] C. Pușcașu , S. Negreș , C. E. Zbârcea , et al., “Evaluating the Antihyperalgesic Potential of Sildenafil–Metformin Combination and its Impact on Biochemical Markers in Alloxan‐Induced Diabetic Neuropathy in Rats,” Pharmaceuticals 17, no. 6 (2024): 783. 10.3390/ph17060783 PMC11206800 38931450

[38] M. Mansouri , B. Naghizadeh , and B. Ghorbanzadeh , “Sildenafil Enhances the Peripheral Antinociceptive Effect of Ellagic Acid in the Rat Formalin Test,” Indian Journal of Pharmacology 46, no. 4 (2014): 404–408. 10.4103/0253-7613.135952 PMC4118533 25097278

[39] I. Melkani , B. Kumar , S. Panchal , et al., “Comparison of Sildenafil, Fluoxetine and Its Co‐Administration Against Chronic Constriction Injury Induced Neuropathic Pain in Rats: An Influential Additive Effect,” Neurological Research 41, no. 10 (2019): 875–882. 10.1080/01616412.2019.1630091 31238812

[40] M. Kaur , A. Singh , B. Kumar , et al., “Protective Effect of Co‐Administration of Curcumin and Sildenafil in Alcohol Induced Neuropathy in Rats,” European Journal of Pharmacology 805 (2017): 58–66. 10.1016/j.ejphar.2017.03.012 28315678

[41] X. Zhu , S. Xie , J. Chen , et al., “Sildenafil Enhances the Therapeutic Effect of Islet Transplantation for Diabetic Peripheral Neuropathy via mTOR/S6K1 Pathway,” International Journal of Endocrinology, no. 1 (2023): 8199029. 10.1155/2023/8199029 PMC10576648 37841556

[42] S. Megat , S. Hugel , S. H. Journée , et al., “Antiallodynic Action of Phosphodiesterase Inhibitors in a Mouse Model of Peripheral Nerve Injury,” Neuropharmacology 205 (2022): 108909. 10.1016/j.neuropharm.2021.108909 34875284

[43] An Open Label . Dose Finding Trial of Viagra for the Treatment of Neuropathic Pain (in Diabetes Mellitus). 2005, https://clinicaltrials.gov/study/NCT00194909.

[44] T. R. Oliveira , T. V. H. Xavier , and T. C. B. S. Mendes , “Uso Do Sildenafil No Controle De Dor Neuropática Após Nefrectomia: Relato De Caso,” Revista Dor 12 (2011): 365–367.

[45] K. Hosseinnejad , T. Yin , J. T. Gaskins , et al., “Lack of Influence by CYP3A4 and CYP3A5 Genotypes on Pain Relief by Hydrocodone in Postoperative Cesarean Section Pain Management,” Journal of Applied Laboratory Medicine 3, no. 6 (2019): 954–964. 10.1373/jalm.2018.026070 31639687

[46] G. Maziero Alves , R. Aires , V. de Souza Santos , et al., “Sildenafil Attenuates Nonsteroidal Anti‐Inflammatory‐Induced Gastric Ulceration in Mice via Antioxidant and Antigenotoxic Mechanisms,” Clinical and Experimental Pharmacology and Physiology 48, no. 3 (2021): 401–411. 10.1111/1440-1681.13414 33020944

